# Ovarian Hormones Mediate Changes in Adaptive Choice and Motivation in Female Rats

**DOI:** 10.3389/fnbeh.2019.00250

**Published:** 2019-11-12

**Authors:** Katie E. Yoest, Jennifer A. Cummings, Jill B. Becker

**Affiliations:** ^1^Department of Psychology, College of Literature, Science, and the Arts, University of Michigan, Ann Arbor, MI, United States; ^2^Department of Psychology, Michigan State University, East Lansing, MI, United States; ^3^Molecular and Behavioral Neuroscience Institute, University of Michigan, Ann Arbor, MI, United States

**Keywords:** sexual behavior, feeding, motivation, ovarian hormones, females

## Abstract

In female rodents, sexual receptivity is coordinated with cyclic changes in the release of gonadal hormones. Increases in estradiol (E) and progesterone (P) during proestrus and estrus not only induce ovulation but also modulate behaviors that increase the likelihood that the female will find a mate and reproduce. This includes changes in receptive behaviors, such as lordosis, as well as changes in appetitive or proceptive behaviors, including motivation. Interestingly, the direction of these changes in motivation is dependent on the type of reward that is being pursued. While induction of sexual receptivity by E and P increases motivation for access to a male, motivation for a palatable food reward is decreased. These concurrent changes may facilitate adaptive choice across the estrous cycle; females bias their choice for sex when fertilization is most likely to occur, but for food when copulation is unlikely to result in impregnation. In order to test this hypothesis, we developed a novel paradigm to measure the motivated choice between a palatable food reward and access to a male conspecific. Ovariectomized, hormone primed females were trained to operantly respond for both food and sex on a fixed interval (FI) schedule. After training, unprimed and primed females were tested in a chamber that allows them to choose between food and sex while still requiring responding on the FI schedule for reach reward. From this we can not only determine the impact of hormone priming on female choice for food or sex, but also how this is reflected by changes in motivation for each specific reward, as measured by the average number of responses made during each fixed interval. Induction of sexual receptivity by hormone priming biases choice toward sex over food and this change is accompanied by an increase in motivation for sex but a decrease in motivation for food. This work provides evidence in support of a novel framework for understanding how the release of ovarian hormones over the course of the estrous cycle modulates adaptive behavioral choice in females by directly assessing motivation *via* operant responding when multiple rewards are available.

## Introduction

All behaving organisms are continually faced with alternative and competing demands from which they must direct behavior in order to enhance their adaptive success. Motivation is a key regulator of these goal-directed behaviors and has been proposed to modulate not only decision-making processes but also the vigor with which these behaviors are executed (Niv et al., 2006). In order for behavior to be appropriately selected based on physiological needs, internal signals for hunger, thirst, and reproductive status have evolved the ability to direct motivation for specific stimuli based on the organism’s internal state (Zardetto-Smith et al., 1993; Balleine, 1994; Dickinson and Balleine, 1994; Salamone et al., 2003; Robinson and Berridge, 2013; Cone et al., 2014; Aitken et al., 2016).

Discrete components of motivated behaviors have classically been categorized as being either appetitive or consummatory (Craig, 1917; Ball and Balthazart, 2008). Importantly, appetitive and consummatory aspects of a behavior can be dissociated, and often are regulated by discrete neural systems (Everitt, 1990; Baldo and Kelley, 2007). Behaviors involved with the consumption of a reward or the act of copulation, in the case of sexual behavior, are considered consummatory behaviors. On the other hand, appetitive behaviors serve to prepare the animal to engage in consummatory behaviors and are inherently more variable as they depend on the circumstances in which the animal engages in the behavior. Appetitive behaviors include behaviors that the animal engages in to locate and obtain rewards.

One example of a motivated behavior, for which the neural circuitry underlying both appetitive and consummatory components have been extensively investigated, is sexual behavior in male rats. When presented with a sexually receptive female, male rats will approach and investigate the female, after which they will attempt to mount and intromit (Hull and Dominguez, 2007). Following repeated intromissions and ejaculation, the male then enters the refractory period, during which locomotor activity and interest in the female are suppressed (Beach and Jordan, 1956). The neural circuitry underlying the consummatory aspects of male sexual behavior (e.g., mounting, intromissions, and ejaculations) and the ability of male gonadal hormones (e.g., testosterone) to modulate this circuitry has been well studied. For example, lesions to the medial preoptic area (MPOA) result in significant impairments in consummatory behaviors. Furthermore, testosterone replacement to the MPOA of castrated male rats, who do not normally exhibit sexual behavior, can reinstate mounting, intromissions, and ejaculation (Christensen and Clemens, 1974; Everitt and Stacey, 1987).

However, examination into the appetitive aspects of male reproductive behavior have yielded different results. Training animals to exhibit an operant response to receive access to a reward, like pressing a lever or poking their nose in a hole, is a paradigm that has been used extensively in a variety of fields when it is important to directly evaluate the level of motivation an animal. Importantly, lesions to the MPOA that abolish consummatory sexual behavior in males have no effect on operant responding for access to a sexually receptive female, indicating that the MPOA does not play a role in male sexual motivation (Everitt, 1990). Conversely, lesions to the basolateral amygdala abolish motivated responding for access to a female without altering the consummatory aspects of sexual behavior (Everitt, 1990).

Applying these same principles to the study of female sex behavior has proved to be more challenging. One reason for this disparity in our understanding of male vs. female sexual behavior is that there are several important differences in the expression and regulation of male and female reproductive behaviors. Male sexual behavior is dependent on the presence of the gonadal hormone testosterone, but adult male rats with intact testes are continuously capable of engaging in sexual behavior (Hull and Dominguez, 2007). Males also find sexual activity most rewarding when the male has free access to the female so that he regulates the mating encounter (Martínez and Paredes, 2001). Females, however, require the coordinated sequential release of estradiol and progesterone in order to induce sexual receptivity (Beach et al., 1942) and motivation (Cummings and Becker, 2012), limiting the time during which she will engage in sexual behavior to the time around ovulation. In addition, females only find a sexual encounter rewarding when the rate of mounts, intromissions, and ejaculations are regulated by the female, a pattern of sexual activity known as pacing behavior (Adler and McClintock, 1978; Jenkins and Becker, 2003b).

Substantial research has shown that when given the opportunity, females will actively pace a sexual encounter by running away from the male following a mount, intromission, or ejaculation (Erskine, 1989). Pacing behavior reflects the sensitivity of the female rat to the intensity of cervical stimulation received during an encounter with the male and modulates the female’s response to that stimulation. Female paced mating also allows for the activation of a neuroendocrine reflex that increases the probability of conception (Erskine et al., 1989). During pacing the female will engage in a complex pattern of behaviors that includes ear wiggling, hops and darts, and other general approach behaviors, that serve not only to attract attention from conspecifics but also to hold the male’s attention between intromissions (Adler and McClintock, 1978; Erskine, 1989). Female sexual behaviors are thus crucial for the full display of reproductive behavior, particularly in the context of ethological relevant mating paradigms, and demonstrate that females play an active role in mating (McClintock and Anisko, 1982; McClintock et al., 1982; McClintock, 1984).

The motivational circuitry important for appetitive behaviors in the female may be anatomically dissociable from the neural circuitry that mediates consummatory aspects of sexual behavior, as was demonstrated in the male rat (Everitt, 1990). However, the majority of research on female sexual behavior has used reflexive behaviors such as lordosis quotient, ear wiggling, and the number of hops and darts as indices of female sexual arousal and motivation (Pfaus et al., 1999; Mazzucco et al., 2008). That these behaviors can be elicited by the experimenter rubbing the rump of the female rat when she is hormone primed calls into question their validity as a measure of sexual motivation. This necessitates that more direct measures of motivation be utilized. In support of this idea, SSRI-induced sexual dysfunction, which primarily affects precopulatory and appetitive sexual behaviors in women, is detectable using operant tasks that measure sexual motivation, but not using classic tests of female proceptive behaviors (Uphouse et al., 2015). This highlights the importance of using behavioral paradigms that specifically measure sexual motivation, without the confound of potentially reflexive components of sexual behavior.

Operant paradigms have been applied to the study of female sexual behavior as early as 1961 (Bermant, 1961; Bermant and Westbrook, 1966). In these studies, females were trained to make an operant response for the presentation of a sexually experienced male rat. The experimenters then measured how the magnitude of stimulation received by the female altered the latency to initiate an operant response and found that the response latencies were positively correlated with the magnitude of stimulation. This is consistent with findings that the latency for females to return to a male when pacing sexual behavior varies with the intensity of stimulation (Erskine, 1989). Operant tasks have also been used to measure female preference for a specific mate (French et al., 1972). Using an FR1 schedule of reinforcement, researchers found that females make more responses during estrus compared to diestrus, and that preferences for one mate over another are only apparent when females are sexually receptive. However, while these early studies provide the first evidence that female rats will work for access to a mate, the use of an FR1 schedule does not allow the response rate to be compared without also comparing the consumption of the primary reward.

By allowing the female to control the rate at which the male was introduced, experimenters were allowing the female to pace the mating encounter, even though the phenomenon of paced mating had not yet been formally described (Adler and McClintock, 1978). Additional research has confirmed and extended these findings. Paced mating is rewarding for receptive female rats: they will develop a conditioned place preference following paced mating and will readily work for access to a mate when they are able to pace the rate of copulation (Paredes and Alonso, 1997; Jenkins and Becker, 2003b; Cummings and Becker, 2012). This demonstrates that sexual behavior is both motivating and rewarding for female rats when it occurs under the right conditions, and thus is likely mediated, at least in part, by the neural circuitry underlying motivation for other rewards.

The major brain system underlying motivated behaviors includes the dopaminergic projections from the substantia nigra (SN) and ventral tegmental area (VTA) to the dorsal striatum (DS) and nucleus accumbens (NAc), respectively (Yoest et al., 2014; DiFeliceantonio and Berridge, 2016). Dopamine (DA) cell bodies in the SN and VTA show altered firing in response to salient environmental events and the cues that predict them, including exposure to a novel environment, delivery of unexpected rewards or cues that predict reward, and even aversive stimuli such as aggressive encounters (Horvitz, 2000). These changes in DA cell firing induce changes in DA signaling and downstream activity that correlates with motivation (Bromberg-Martin et al., 2010). Importantly, DA signaling is responsive to homeostatic changes in the animal’s internal state, both through direct effects of peripheral signaling molecules on mesolimbic DA circuitry, as well as through projections from extra-striatal areas involved in maintaining homeostasis (Jerlhag et al., 2006; Cone et al., 2014; Nieh et al., 2016; Woods et al., 2016; Baimel et al., 2017; McHenry et al., 2017). This has been hypothesized to facilitate changes in motivation for specific rewards in accordance with the organism’s physiological or adaptive need.

Ovarian hormones may regulate changes in motivated behavior through effects on DA responsivity. Stimulated DA release in both the DS and NAc is enhanced during proestrus and estrus, when levels of circulating hormones are at their highest (Xiao and Becker, 1994; Thompson and Moss, 1997; Calipari et al., 2017). A large body of work has demonstrated that the ovarian hormone estradiol rapidly enhances stimulated DA release in females but not in males (Yoest et al., 2018). But while the majority of research has focused on the effect of estradiol on the DA system, the induction of many motivated behaviors associated with the ovulatory cycle requires the release of both estradiol and progesterone (Tennent et al., 1980). Thus, treatment with estradiol alone potentiates stimulated DA release, and treatment of estradiol-primed animals with progesterone further enhances stimulated striatal DA release, above the effects of estradiol alone (Dluzen and Ramirez, 1984; Becker and Rudick, 1999). Interestingly, the effect of progesterone on DA release is biphasic, 30 min–4 h following progesterone treatment DA release is enhanced, but DA release is attenuated 24 h after progesterone administration (Dluzen and Ramirez, 1984). This time course coincides with the maximal induction of sexual receptivity following hormone priming, as well as an increase in female sexual motivation (Cummings and Becker, 2012). This suggests that modulation of striatal DA release around the time of ovulation is important for specific components of sexual behavior, but the role of hormone-mediated changes in DA release during periods of sexual receptivity has not yet been examined.

Research in male rats has implicated DA in copulatory ability as well as appetitive components of sexual behavior. DA levels in both the NAc and striatum is increased in response to a sexually receptive female and subsequent copulation, and this rise in DA is seen regardless of sexual experience, indicating that it is not a consequence of learning (Wenkstern et al., 1993; Pfaus et al., 1995; Robinson et al., 2001). Additionally, pharmacological inactivation of DA receptors in male rats increases, while administration of the DA agonist amphetamine decreases, operant responding for access to a female latency to mount and intromit in male rats (Everitt, 1990). Importantly, extracellular DA levels in striatum and NAc in the female increase during sexual behavior only when copulation occurs at the female’s preferred interval (Mermelstein and Becker, 1995; Pfaus et al., 1995; Becker et al., 2001; Jenkins and Becker, 2003a). The greatest increase in DA release is seen prior to the male’s intromission, and is not due to sensory stimulation or non-copulatory social interaction, indicating that DA is involved in anticipation of sexual behavior, rather than the sensorimotor aspects of sexual behavior (Jenkins and Becker, 2003a).

The conditioned place preference induced by mating may also be DA dependent. In female hamsters, administration of a D2 DA receptor antagonist prevents the formation of a conditioned place preference (Meisel et al., 1996). However, other groups have found that DA antagonists do not block place preferences induced by female paced mating, but instead that the opioid system regulates mating induced conditioned place preference in female rats (Paredes and Martínez, 2001; García Horsman and Paredes, 2004). While this may be due to species-specific regulation of sexual reward, this discrepancy may also be explained by methodological differences. When copulation occurs in the conditioning chamber, the place preference is sensitive to DA receptor manipulations (Meisel et al., 1996). Alternatively, when the female is placed into the conditioning chamber immediately following copulation, the formation of the place preference is dependent on opioid transmission (Paredes and Martínez, 2001; García Horsman and Paredes, 2004). Taken together with findings that DA dynamically increases during female paced mating, this may indicate that DA mediates active, motivated components of female sexual behavior, while the opioid system is involved in post-copulatory components of the rewarding aspects of sexual behavior.

Increases in hormones during proestrus and estrus that induce ovulation and enhance striatal DA release also modulate behaviors that increase the likelihood that the female will find a mate and reproduce (Fessler, 2003). Changes in receptive behaviors (e.g., lordosis), proceptive behaviors (e.g., ear wiggling), and other aspects of appetitive behavior (including motivation) are modulated by ovarian hormones. Importantly, while estradiol and progesterone appear to non-specifically enhance stimulated DA release, the effect of these hormones on motivation is dependent on the type of reward that is being pursued. Increases in estradiol and progesterone lead to enhanced motivation for access to a mate but decrease motivation for food (Cummings and Becker, 2012; Richard et al., 2017).

The adaptive benefit of coordinating sexual behaviors with ovulation is quite clear. Copulatory behaviors are accompanied by necessary danger: risk of predation during mate-seeking, injury due to the copulatory act itself, or infectious disease (Daly, 1978). Therefore, females that are not in estrus are not motivated to find a mate and will actively reject male advances (Hardy, 1972; Cummings and Becker, 2012). However, estrogens, and to a lesser extent, progesterone, have also been implicated in the regulation of feeding behavior and motivation for food. Food intake and body weight are both decreased around the time of ovulation (Blaustein and Wade, 1976). Removal of ovarian hormones through ovariectomy increases body weight by increasing food intake, and cyclic estradiol replacement in ovariectomized females restores normal feeding patterns and body weight (Wade, 1972; Asarian and Geary, 2002). The effects of estradiol on the neural circuitry regulating food intake are diffuse; estradiol has been shown to interact with both orexigenic and anorexigenic peptides in a number of different brain areas (Eckel et al., 2002; Clegg et al., 2006; Brown and Clegg, 2010; Santollo et al., 2011; Mela et al., 2016). While estradiol has been shown to be sufficient for the regulation of consummatory feeding behavior in females, progesterone may also modulate feeding. Progesterone is positively correlated with feeding in women, and others have speculated that progesterone may inhibit the anorexigenic effects of estradiol on feeding (Wade, 1972; Yu et al., 2011; Roney and Simmons, 2017).

These changes in feeding behavior around the time of ovulation are less obviously adaptive than changes in sexual behavior. Copulation and reproduction are energetically costly, and would be expected to require greater food intake, but females show decreased food intake and motivation for food around the time of ovulation (Asarian and Geary, 2006; Richard et al., 2017). In order to explain this paradoxical change in feeding behavior, researchers have speculated that changes in feeding behavior serve to reduce the amount of time and energy that animals dedicate toward obtaining and consuming food, thus increasing the amount of time available for mate-seeking and reproductive activities (Fessler, 2003; Schneider et al., 2013). Although this hypothesis has powerful explanatory potential as an ultimate explanation of animal behavior, it remains untested within experimental settings.

Similarly to sexual behavior, research on hormonal regulation of feeding behavior in female rats has focused on changes in consummatory aspects of food intake (Rivera and Stincic, 2018). Few studies have evaluated the direct effect of estradiol on motivation for food, even though many of the signaling molecules regulated by estradiol have been shown to regulate motivated feeding (Cone et al., 2014; Olarte-Sánchez et al., 2015; Stouffer et al., 2015; van der Plasse et al., 2015; Hayes and Schmidt, 2016). The limited work that has been done has shown that motivation for palatable food reward is reduced during proestrus and estrus, an effect that is mediated by estradiol acting directly on the VTA (Richard et al., 2017). Further, repeated treatment with estradiol potentiates the ability of GLP-1 to attenuate motivation for food *via* ERα (Richard et al., 2016).

Even less work has been done to investigate the role of progesterone in feeding behavior. Progesterone is positively correlated with feeding in women, and others have speculated that progesterone may inhibit the anorexigenic effects of estradiol on feeding (Wade, 1972; Yu et al., 2011; Roney and Simmons, 2017). The effect of progesterone on motivation for food is unknown.

In addition to the paucity of work investigating the effect of ovarian hormones on motivated behaviors, what work that has been done has evaluated motivation when only one reward is available. This context strongly contradicts the natural environment, where organisms are continuously faced with opportunities for alternate rewards. In the natural environment, decisions must be made based on the value of the reward, likelihood of receiving the reward, as well as the animal’s internal state (Carr, 1996; Niv et al., 2006; Porter-Stransky et al., 2013; Aitken et al., 2016; Bach and Dayan, 2017). Often, pursuit of one reward precludes the ability to earn other available rewards, requiring the animal to not only decide which reward they should pursue but also which reward they must forgo.

To this end, our lab has developed an operant paradigm that quantitatively measures the female’s motivation to obtain access to a sexually active male, while allowing the female to actively pace the sexual encounter (Cummings and Becker, 2012). In this paradigm, females respond on a fixed interval schedule for access to a sexually experienced male conspecific that is tethered in an adjacent chamber. Importantly, the use of the fixed interval schedule allows us to dissociate the effects of ovarian hormones on consummatory aspects of sexual behavior from effects on motivation by quantifying the number of responses the female makes to gain access to the male within a fixed period of time. We have adapted this paradigm to measure concurrent changes in motivation for both food and access to a mate when multiple rewards are available. Therefore, the current experiment aimed to evaluate whether concurrent changes in motivation for food vs. a mate are able to facilitate motivated choice during periods of sexual receptivity. Ovariectomized (OVX) female rats were trained to respond to both food and a mate simultaneously. During testing, animals were able to choose between the two rewards, after which the number of responses animals made for each reward was used as an indicator of their motivation. Thus, the present study demonstrates that increases in ovarian hormones associated with induction of sexual receptivity directs both choice of and motivation for food and a mate in female rats.

## Materials and Methods

### Animals

Ten female Long Evans rats 50–55 days of age and 15 proven breeder stimulus males (Charles River Breeding Laboratory; Portage, MI, USA) were maintained on a 14:10 L:D cycle (lights of at 13:00 h) and housed in same-sex pairs in large laboratory cages (Allentown NextGen 1800; Allentown, NJ, USA) with ad libitum access to water and phytoestrogen free rat chow (2017 Teklad Global, 14% protein rodent maintenance diet, Harlen rat chow; Harlan Teklad, Madison, WI, USA). All procedures were carried out in accordance with the National institutes of Health Guidelines on laboratory animal use and care, using a protocol approved by the University of Michigan Institutional Animal Care and Use Committee. Experimental animals were OVX as previously described (Cummings et al., 2014). Vaginal lavage samples were collected daily starting 10 days after surgery in order to verify absence of estrous cycle.

### Drug Preparation and Hormone Priming

Estradiol benzoate (EB; Sigma Aldrich, MO, USA; 5 μg/0.1 ml) and P (Sigma Aldrich, MO, USA; 500 μg/0.1 ml) were administered subcutaneously in order to induce sexual receptivity in OVX females. Both hormones were emulsified in peanut oil and stored at room temperature for the duration of use. EB was administered at 13:00 h for 2 days, followed by P at 1,000 h on the third day. Animals were considered fully hormone primed and sexually receptive 4–6 h after P administration.

### Operant Task

**Figure 1:**
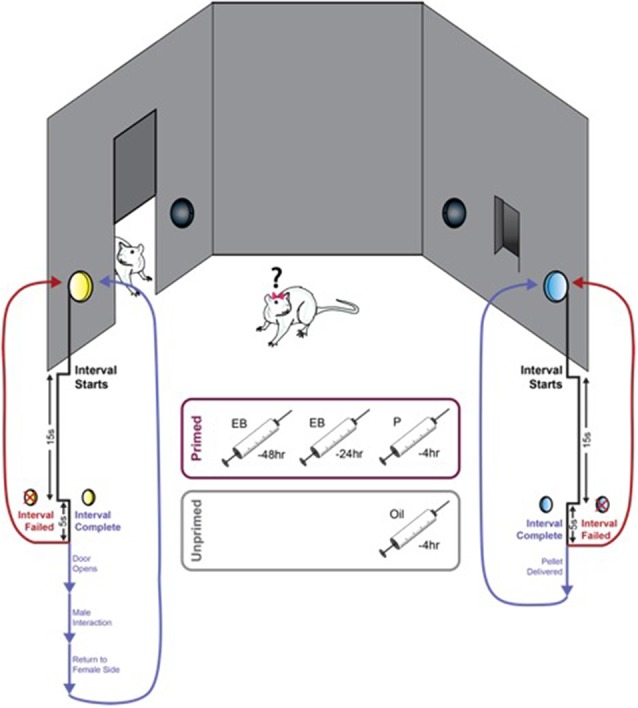
An operant paradigm for concurrent measurement of motivation for food and sex. Female rats were trained to respond for both food and sex on a 15 s fixed-interval (FI15) schedule. Activation of either active port initiated an FI15 for that specific reward (food or sex), and animals could only respond for access to the initially selected reward for the duration of the trial. Completion of the FI15 on the active port located adjacent to the food trough resulted in the delivery of a palatable food pellet (45 mg banana flavor, BioServ, Flemington, NJ, USA) accompanied by presentation of a discrete light cue for 1 s. Completion of the FI15 on the active port located adjacent to the door leading to the other chamber resulted in the door opening and presentation of a different light cue for 1 s. The door remained open until the female crossed into the male side to interact with the male and then closed upon her return to the female side. Failure to complete the FI15 did not result in reward delivery, and the next response made on either port would initiate a new trial.

All training and testing took place in custom-built operant pacing chambers (Figure 1). Control of the apparatus and video recording and analysis was performed using AnyMaze (Stoelting, Wood Dale, IL, USA). Two compartments within the chamber were separated by a horizontal sliding door. The larger compartment contained a tethered stimulus male (Male Side). The smaller of the two compartments (Female Side) was outfitted with four nose poke ports. Two ports were located on the wall adjacent to the sliding door and served as response elements (active and inactive) to open the door. The additional two ports were located on the wall opposite the door spaced around the food tray in which a palatable food pellet (45 mg banana flavor, BioServ, Flemington, NJ, USA) would be delivered. Responding on the active port for each reward resulted in activation of a discrete light cue located directly above the port and initiation of the FI15. During this interval, all responses were counted and resulted in presentation of the cue light but had no other consequence. This second-order schedule, in which responding during the fixed interval is maintained by presentation of the reward-paired light cue, allows for a distinction to be made between the primary reinforcement value of the reward and the incentive motivational value of the cue. This is particularly important when using the number of responses during a fixed interval schedule as the index of motivation for sex as the primary reward (Everitt, 1990). The first response made within 5 s of the conclusion of this 15 s interval resulted in either delivery of a single food pellet or activation of the sliding door to allow access to the second compartment. If the female did not make a response within this 5 s window, the interval was failed, and no rewards were delivered until the animal initiated and completed a new trial. Importantly, although both rewards were concurrently available, initiation of the FI15 for one reward precluded responding to earn the other reward until the initial FI15 was either completed or failed. Any responses made for the other reward during this window were counted but did not result in activation of the cue light and could not initiate a new trial or earn the reward. Thus, animals must first choose between the two available rewards, and then sustain responding for this choice until the end of the 15 s interval in order to be rewarded.

### Training Paradigm and Schedule

Animals were initially trained to respond for each reward separately. All training sessions lasted 30 min and animals had no more than one training session per day. Animals started training on a fixed-ratio (FR) 1 schedule, during which every response on the active port resulted in delivery of the respective reward. Once animals made at least 10 active responses during training for sex, and 20 active responses during training for food, the FR requirement was increased to five. The FI schedule was introduced after animals mastered the FR5 (same criterion as FR1). Animals continued training on the FI15 for each reward separately for 1 week, at which point they started training on the concurrent FI15 schedule. At this point, animals were trained twice a week, once when unprimed and once when primed. Training on the concurrent FI15 continued for 3 weeks. Eight out of 10 animals reached stable levels of responding after this point. The two animals that failed to successfully learn the task were excluded from subsequent analyses.

### Testing Schedule

During the week of testing, animals were tested once when primed and once when unprimed. The order of testing was counterbalanced across animals and animals were always tested with a novel stimulus male. Four hours prior to testing (10:00 h), animals were given a single subcutaneous injection of either P, if they had been primed with EB, or oil, if they were unprimed. At this time, food hoppers were removed from the home cage and animals were lavaged to verify hormonal status. Although we have previously found that female rats will perform an operant task for food reward even when fed ad libitum, the removal of food from the home cage and the decision to test females at the beginning of the dark phase when food intake is at its highest were both intended to increase the likelihood that females would work for the palatable food reward (Rosenwasser et al., 1981; Perry et al., 2013). At 02:00 h, animals were transported to the testing room. Testing sessions lasted 30 min, after which animals were returned to their home cage.

### Video Scoring

Behavioral video was scored offline by an observer blind to the animal treatment group. Videos were analyzed to verify the amount of time animals spent in each chamber during testing, as well as to determine which components of the apparatus animals engaged with. All durations were normalized to the total amount of time in the chamber prior to analysis. Finally, sexual behavior was scored in order to account for the effect of the male’s behavior on female sexual motivation.

### Statistical Analysis

Group comparisons were performed using GraphPad Prism v7.0a (GraphPad, San Diego, CA, USA). Shapiro–Wilk normality tests were used to test for normal distributions. The effect of hormone priming on discrete variables was analyzed using paired *t*-tests or Wilcoxon matched-pairs signed-rank tests when data violated the assumption of normality. Interactions between hormone priming and other variables, e.g., trial type, were analyzed using two-way repeated-measures ANOVA with Holm–Sidak *post hoc* tests (Supplementary Table S1). The effect of ejaculation on motivation for sex in hormone primed animals was analyzed using one-way ANOVA. Changes in responding for food pellets were analyzed within treatment groups by linear regression to determine if the slope of the line was significantly different from zero. Data are presented as mean ± SEM except where stated otherwise.

## Results

### Induction of Sexual Receptivity Alters Preference for Food vs. Sex

**Figure 2:**
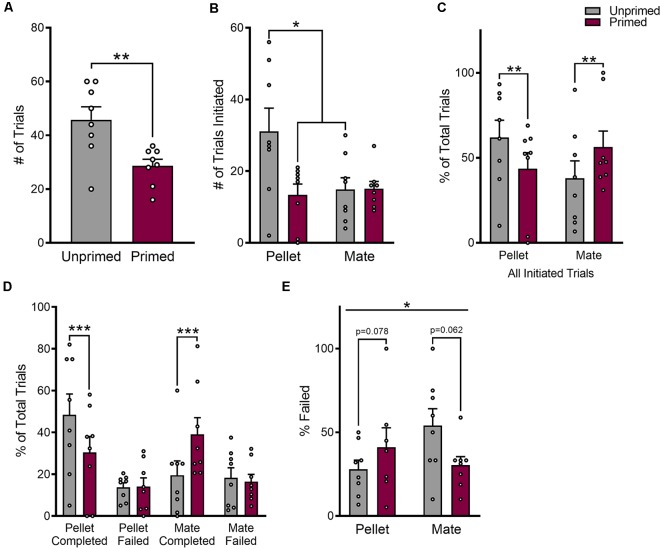
Reproductive status biases choice for food vs. sex. Hormone treatment reduced the total number of trials that animals initiated **(A)**, specifically by reducing the number of pellet trials **(B)**. The proportion of mate or pellet trials that animals initiated was altered following hormone treatment **(C)**. Animals initiated more mate trials when hormone primed than when unprimed, and more pellet trials when unprimed than when hormone primed. Changes in the proportion of pellet or mate trials that animals initiated were driven by increases in completed trials, without altering the total number of trials that animals failed **(D)**. Although the total number of failed trials remained unchanged, changes in the corresponding number of completed trials resulted in a significant interaction between hormone treatment and trial type on the proportion of trials that animals failed **(E)**. Data are shown as mean ± SEM. Data points represent within session means for individual animals, *n* = 8, within subject design. *p < 0.05, **p < 0.01, ***p < 0.001.

The number of trials that a female initiated in pursuit of each reward was used as an indicator of the primary reinforcement value of each reward. There was a significant main effect of hormone priming on the number of trials that animals initiated (*F*_(1,7)_ = 19.27, *p* < 0.01), that differed between food and mate trials (*F*_(1,7)_ = 7.30, *p* < 0.05). As shown in Figure 2B, hormone primed animals initiated fewer food trials than unprimed animals (*p* < 0.05), but a similar number of mate trials overall (*p* = 0.96). However, unprimed animals also initiated a greater number of trials overall (*t*_(7)_ = 4.53, *p* < 0.01; Figure 2A). Therefore, in order to account for differences in the total number of trials that animals initiated, we normalized the number of mate vs. pellet trials to the total number of trials initiated during each session. This normalized value reflects the preference for one reward over the other. After normalizing, there was still a significant effect of hormone priming on the number of mate vs. food trials that animals initiated relative to the total number of trials initiated that differed between food and mate trials (*F*_(1,14)_ = 22.20, *p* < 0.001; Figure 2C). Hormone primed animals initiated a smaller proportion of food trials (*p* < 0.01) but a greater proportion of mate trials (*p* < 0.01) than unprimed animals, indicating that hormone priming does indeed bias choice toward a sexual partner and away from a palatable food reward.

Hormone priming specifically increased the number of completed food (*p* < 0.001) or mate (*p* < 0.001) trials, without altering the number of failed trials for either reward (food: *p* = 0.94, mate: *p* = 0.88; Figure 2D). After further controlling for the total number of trials initiated in pursuit of each reward, we found that hormone priming significantly altered the proportion of failed trials, but this effect was again dependent on the reward that was being pursued (*F*_(1,27)_ = 4.88, *p* < 0.05; Figure 2E).

**Figure 3:**
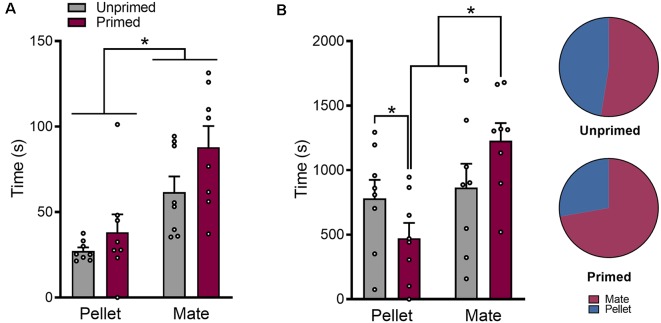
Hormone treatment alters the amount of time animals spend in pursuit of each reward. **(A)** Mate trials are on average longer than pellet trials, and trial length for both rewards is increased in after hormone priming. **(B)** The total amount of time that animals spent engaged in mate trials was increased following hormone priming, which was accompanied by a decrease in the amount of time spent engaged in pellet trials. Data are shown as mean ± SEM. Data points represent within-session means for individual animals, *n* = 8, within-subject design. *p < 0.05.

Pre-treatment with ovarian hormones also altered the amount of time that animals spent engaging in food or mate-seeking. There was a significant effect of hormone priming on the average duration of food and pellet trials (*F*_(1,7)_ = 11.43, *p* < 0.05) where all trials were longer when animals were hormone primed (Figure 3A). In addition, mate trials were longer than pellet trials overall (*F*_(1,7)_ = 7.11, *p* < 0.05) but this effect did not differ by hormone treatment (*F*_(1,7)_ = 0.76, *p* = 0.41; Figure 3A).

When looking at total duration of time animals spent engaging in either food-seeking or mate-seeking, a measure that accounts for both differences in the number of trials animals initiated as well as the duration of each trial, there was a significant interaction between trial type and hormone treatment (*F*_(1,7)_ = 25.16, *p* < 0.01). As shown in Figure 3B, unprimed animals spent a similar amount of time engaged in mate and pellet trials (*p* = 0.40), while in primed animals the total amount of time spent in mate trials was significantly greater than the amount of time spent in pellet trials (*p* < 0.001).

### Hormone Priming Alters Motivation for Food vs. Sex

**Figure 4:**
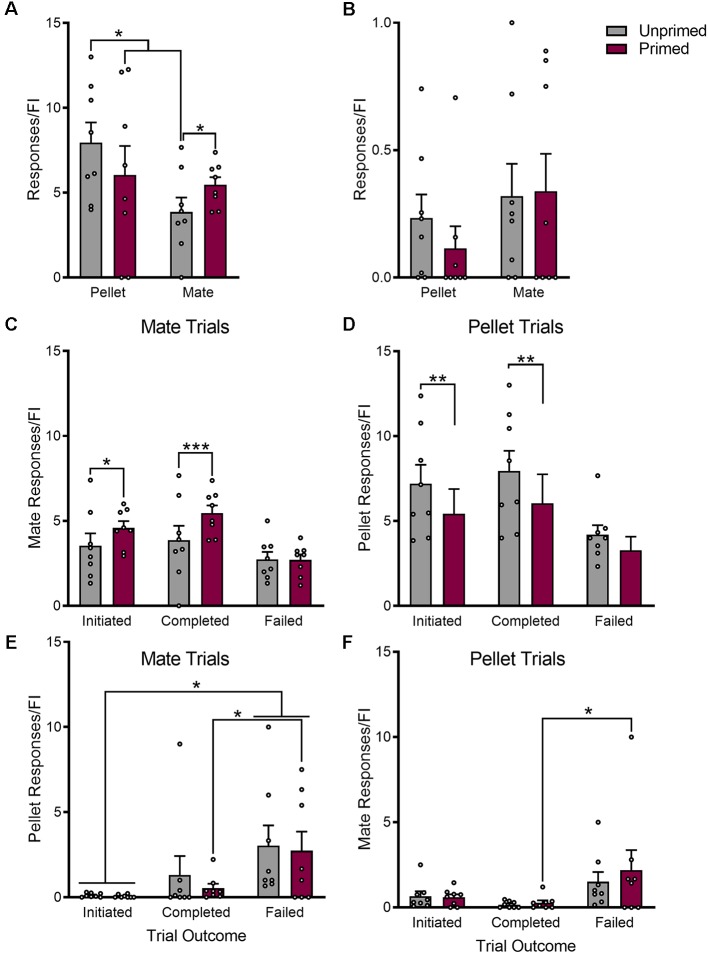
Hormone priming increases motivation for sex while simultaneously decreasing motivation for palatable food reward. The effect of hormone priming on motivated responding is dependent on the reward being pursued **(A)**. Unprimed animals show greater motivation for food than for access to a mate. Induction of sexual receptivity by hormone priming reduces motivation for food, but increases motivation for access to a mate, resulting in similar levels of motivated responding for both rewards. The effect of hormone priming on motivated responding is not mediated by changes in overall locomotor behavior, as there was no effect of hormone priming on the number of responses made on the inactive ports **(B)**. Hormone priming specifically increased responding during completed mate trials, without changing the number of active mate responses during failed trials **(C)**. Similarly, hormone priming only reduced responding for pellet during completed pellet trials, but not during failed pellet trials **(D)**. Although responding for the active reward was not altered during failed trials, hormone primed animals made more responses for the inactive reward during failed trials than completed trials during both mate **(E)** and pellet **(F)** trials. Both primed and unprimed animals made more responses for the alternate reward during failed trials when compared to all trials that were initiated. Data are shown as mean ± SEM. Data points represent within-session means for individual animals, *n* = 8, within-subject design. *p < 0.05, **p < 0.01, ***p < 0.001.

The number of responses that females made during each fixed interval trial were used as a quantitative measure of incentive motivation for each reward. In addition to altering which reward females chose more frequently, hormone priming increased motivation for sex (*p* < 0.05), while simultaneously reducing motivated responding for pellets (*p* < 0.05; Figure 4A). This effect of hormone priming on responding was dependent on whether or not the trial was rewarded (Pellet trials: *F*_(2,14)_ = 8.10, *p* < 0.01; Mate trials: *F*_(2,14)_ = 19.18, *p* < 0.0001). Hormone priming only reduced motivated responding for sex when the trial resulted in the delivery of reward (*p* < 0.001), and not during failed trials (*p* = 0.91; Figure 4C). The same was true during pellet trials (Figure 4D); where primed animals made fewer responses than unprimed animals during completed trials (*p* < 0.01), but not during failed trials (*p* = 0.07).

Although hormone treatment did not alter motivated responding for the initially chosen reward during failed trials, there were important differences in responding for the alternate reward during failed trials (Pellet trials: *F*_(2,14)_ = 4.40, *p* < 0.05; Mate trials: *F*_(2,14)_ = 4.67, *p* < 0.05). Animals made significantly more mate responses during failed pellet trials than completed pellet trials (*p* < 0.05), but only when they were hormone primed (Figure 4F). Interestingly, hormone primed animals also made more pellet responses during failed mate responses (*p* < 0.05; Figure 4E).

### Motivation for Access to a Mate Is Reduced Following Ejaculation

**Figure 5:**
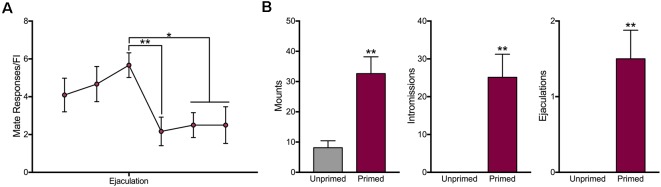
Motivation for access to a mate decreases following ejaculation in sexually receptive female rats. **(A)** Motivation for access to a mate was attenuated during three trials following ejaculation. **(B)** As expected, only sexually receptive animals engaged in sexual behavior. Data are shown as mean ± SEM. For panel **(A)**, data are shown as mean *n* = 12 across eight animals. For panel **(B)**, data shown are mean ± SEM, *n* = 8, within-subject design. *p < 0.05, **p < 0.01.

Animals were only sexually receptive following hormone treatment (Figure 5B). Hormone treated animals received significantly more mounts (*t*_(7)_ = 4.02, *p* < 0.01), intromissions (*t*_(7)_ = 4.12, *p* < 0.01), and ejaculations (*t*_(7)_ = 3.97, *p* < 0.01). In order to determine the effect of male ejaculation on sexual motivation, we compared the average number of mate responses during the trials leading up to and following ejaculation. As shown in Figure 5A, ejaculation significantly reduced motivation for access to a mate (*F*_(5,57)_ = 3.487, *p* < 0.01). Animals decreased responding during the three trials following ejaculation (Trial 1: *p* < 0.01; Trial 2: *p* < 0.05; Trial 3: *p* < 0.05).

### Motivation for Food Increases Over the Test Session

**Figure 6:**
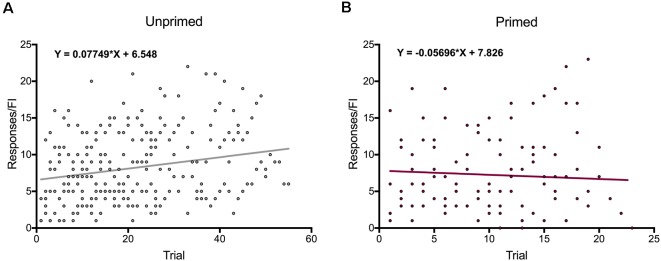
Changes in motivation for food over time. **(A)** Motivation for food increases significantly in over the course of each session in ovariectomized female rats. **(B)** Primed females do not increase their motivation for food from the beginning to the end of each session. Data represent the number of responses during individual trials, *n* = 56 across eight animals for unprimed trials and *n* = 23 across eight animals for primed trials.

In order to determine if animals altered their motivation for food over the course of the session, we plotted the number of responses females made during pellet trials as a function of trial number. We found that overall the number of responses animals made during the session increased over time (slope compared to zero: *F*_(1,54)_ = 15.91, *p* < 0.001), indicating that satiety is not influencing motivation for food during the test session. Interestingly, when animals were grouped by hormone treatment, there was a significant increase in the number of active pellet responses unprimed animals (Figure 6A) made during pellet trials (slope compared to zero: *F*_(1,54)_ = 17.96, *p* < 0.0001), but no change in responding over time in hormone-treated animals (slope compared to zero: *F*_(1,21)_ = 0.41, *p* = 0.53; Figure 6B). This indicates ovariectomized females increase their motivation for food over the course of the session, but not after hormone priming.

### Hormone Priming Biases Where Animals Are Located in the Chamber

**Figure 7:**
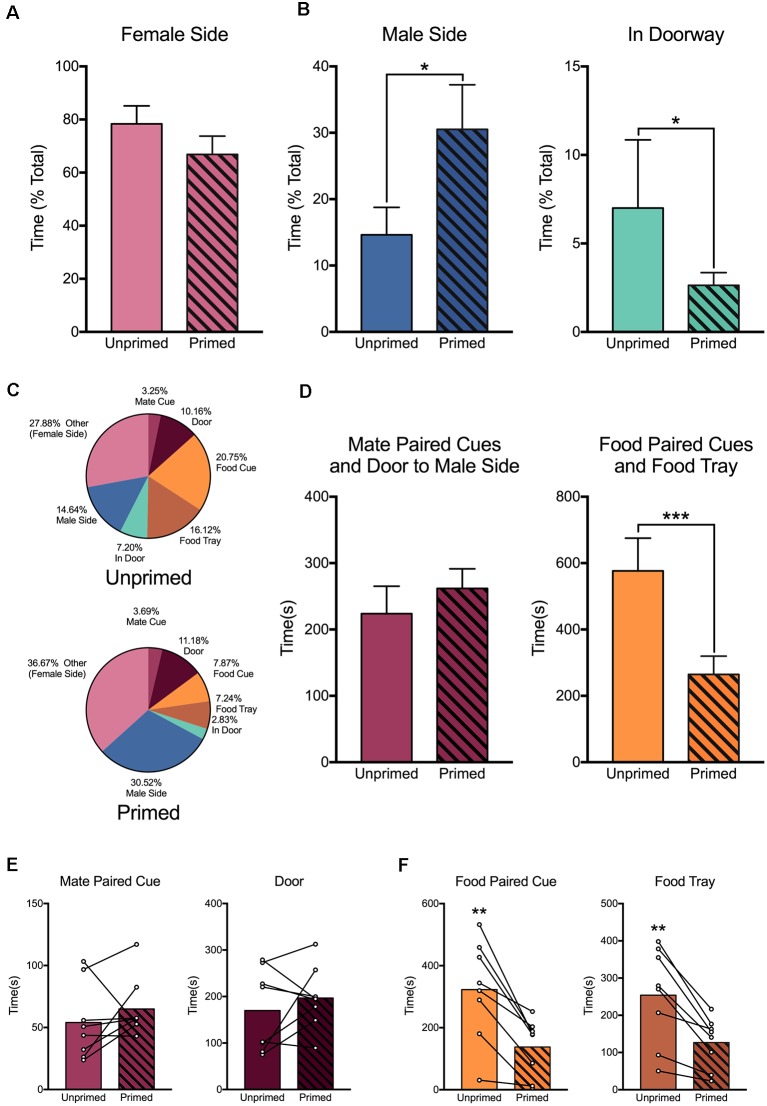
Hormone priming alters the behavior of female rats within the chamber. **(A)** Females spent a similar amount of time in the female (instrumental) compartment regardless of hormone treatment. **(B)** However, when females were given access to the male chamber, hormone primed animals spent more time with the male, while unprimed animals spent more time in the doorway, out of reach of the male. **(C)** Hormone priming altered the distribution of time that animals spent engaging in various aspects of the task. **(D)** Hormone primed animals spent a similar amount of time engaging with the mate paired cue, active mate port, and door, but reduced the amount of time they spent engaging with the food paired cue, active food port, and food trough. This was true when considering the amount of time animals engaged with each aspect of the apparatus individually for both mate **(E)** and food **(F)** paired cues. For panels **(A,B,D)**, data are shown as mean ± SEM, *n* = 8, within-subject design. Mean values are used in panel **(C)**. In panels **(E,F)**, bars represent mean values and data points for individual values, connected to indicate within-subject changes. *p < 0.05, **p < 0.01, ***p < 0.001.

Females were willing to work for access to a mate regardless of hormone treatment. However, receptive and non-receptive animals differed in their behavior once they gained access to the male chamber (Figure 7B). Receptive females spent a greater proportion of time in the male side (*t*_(7)_ = 2.37, *p* < 0.05). Alternatively, when animals were not sexually receptive, they spent more time in the door to the chamber, where they could see the male but he could not physically interact with them (W_8_ = −34, *p* < 0.05). Although females spent a comparable amount of time on the female side regardless of hormonal status (*t*_(7)_ = 1.357, *p* = 0.22; Figure 7A), there were important differences in how they directed their focus within the operant chamber (Figure 7C).

Non-primed females spent more time oriented toward and engaging with the pellet nose poke hole, cue, and food tray than primed females (*t*_(7)_ = 5.42, *p* = 0.001; Figure 7D, right panel). This was also true when we measured attention toward the food associated cues (*t*_(7)_ = 4.86, *p* < 0.01) or food tray alone (*t*_(7)_ = 4.77, *p* < 0.01; Figure 7F). However, there was no effect of hormone treatment on the amount of time that animals spent engaging with the mate nose poke hole, cue, or door (*t*_(7)_ = 1.00, *p* = 0.35; Figure 7D, left panel, Figure 7E). This indicates that hormone primed females, when they are not actively attending to the task, are engaging in some third behavior, presumably waiting for the desired time period between intromissions.

## Discussion

Scholars have long speculated that seemingly paradoxical reductions in food intake and body weight during periods where energetic demand is increased and food remains freely available serve to decrease the likelihood that feeding behaviors will disrupt other, more important, activities (Mrosovsky and Sherry, 1980). One such example that has been the subject of much research is the peri-ovulatory decrease in food consumption seen in most female mammals (Tarttelin and Gorski, 1971; Wade, 1972, 1975; Fessler, 2003; Asarian and Geary, 2006). However, while the proximate mechanisms underlying the effects of the ovulatory cycle on food intake are well understood, enquiry into the ultimate or adaptive purpose of these changes remains mostly speculative. Here, we describe experimental evidence that administration of EB + P to induce sexual receptivity in female rats simultaneously biases both choice and motivation for sex over food and propose an adaptive framework for the interpretation of these behavioral changes.

### Induction of Sexual Receptivity Biases Choice Between Sex and Food

OVX female rats trained to respond on a concurrent FI operant paradigm for both food and sex show a bias toward choice of food reward over access to a sexually experienced male conspecific. This is indicated by both the number of trials that animals initiate in pursuit of the palatable food reward, as well as a shift in the proportion of pellet vs. mate trials. Hormone priming that induces sexual receptivity reduces the number of pellet trials that animals initiate, therefore shifting the proportion of pellet vs. mate trials toward a preference for pursuit of access to the male, but does not increase the total number of mate trials that animals initiate. This may be due to differences in the average length of each trial, and particularly in the amount of time that animals spend with the male after gaining access to the male compartment. Indeed, hormone-treated animals spent more time engaged in mate-seeking and copulation compared to food-seeking, as well as when compared to the amount of time unprimed animals spent engaged in mate-seeking behaviors. Taken together, this suggests that measurement of the raw number of times the female will attempt to gain access to the male is a poor indicator of her sexual motivation and that other measures must also be considered.

### Parsing Sexual vs. Social Motivation

Rats are social animals and will work to gain access to a same-sex conspecific even over drug reward (Venniro et al., 2018). Thus, it is not surprising that non-receptive females will still engage with the operant task in pursuit of a social reward. However, when non-receptive females do gain access to the male, their behavior differs in several important ways. When hormone treated, females engage in sexual behavior (Figure 4B) and spend more time with the male and less time in the doorway between the two sides of the cage (Figure 5B). Unprimed females spend comparatively less time with the male and instead will remain in the doorway where they can see the male but are out his reach and cannot physically interact with him. This further indicates when unprimed females respond for access to the male, they are not doing so in pursuit of sexual reward, but instead for social reward or general novelty seeking. In addition, motivated responding on the fixed interval schedule was sensitive to trial-by-trial changes in sexual motivation. Motivation for access to a mate was attenuated during the male’s post-ejaculatory refractory period. This is consistent with previous work showing that the latency for females to respond for access to a male is increased following ejaculation compared to following a mount or intromission (Bermant, 1961). If operant responding for access to the male was driven by social motivation, we would not expect response rates to be sensitive to changes in sexual satiety.

We used a second-order schedule, in which the reward contingent light cue gains incentive motivational properties, in order to maintain responding during the fixed interval leading up to the presentation of the primary reinforcer. It is possible that the light cue was able to drive responding for access to the male independently of changes in motivation for the actual sexual reward. However, previous work has demonstrated that rats will alter their cue-motivated operant responding in response to changes in homeostatic state (Robinson and Berridge, 2013). This suggests that cue-driven responding is not independent of motivation for the primary reward, but instead reflect dynamic changes in motivation.

### Induction of Sexual Receptivity Has Reward-Specific Effects on Motivation

The number of responses that a female made on the FI schedule can be used as an indicator of her motivation for each reward without being confounded by changes in the number of rewards received. As such, females were more motivated for a palatable food pellet when unprimed, but more motivated for access to a mate when sexual receptivity was induced. Interestingly, this shift in motivation for sex vs. food appears to equalize motivation for the two rewards during periods of sexual receptivity. When females are not sexually receptive, they show greater motivation for food, as opposed to the access to the male. After hormone priming, motivation for food decreases, while motivation for sex increases, leading to a comparable level of responding for both rewards in hormone-treated animals.

This is somewhat surprising, as one would expect that sexually receptive animals would show greater operant responding for sex compared to food. There are a number of potential explanations for this difference. One explanation is that motivation for food remains high because it is still adaptive for females to be motivated for food, even when sexually receptive. Even sated animals will respond to palatable food reward, a strategy that is clearly beneficial in unpredictable environments where food availability is sporadic. Indeed, estradiol alters food intake by decreasing meal size without altering the meal frequency by enhancing the effects of satiety hormones (Blaustein and Wade, 1976; Eckel et al., 2002; Santollo et al., 2007; Brown and Clegg, 2010; Maske et al., 2017). This makes sense within the adaptive explanation that has been proposed—specifically enhancing satiety mechanisms ensures that females will not overlook opportunities to eat but instead spend less time eating during each bout in order to return to the important business of mate-seeking and reproduction. In support of this, we found that females generally increased their motivation for food over time, but this did not happen when they were hormone primed. This does not indicate satiety specifically, as primed females do not show decreased motivation for food over time, but does suggest that there is an effect of hormone treatment on how motivation for food changes over the course of the session, where normal increases in motivation for palatable food reward are blunted in primed females. Alternatively, although animals in the current experiment were not food deprived at any point, we did mildly restrict access to food by removing their food from the home cage 4 h prior to testing, and 3 h prior to the start of the dark cycle. Ad lib fed female hamsters show a strong bias toward sex when both food and males are freely available, which is reversed following food deprivation (Schneider et al., 2007). It is possible that even the marginal food restriction used in the current experiment prevents any further decrease in motivation for food in hormone-treated female rats.

### Effects of Hormone Priming on Task Performance

The effect of hormone treatment on choice between food and sex was specifically driven by an increase in the number of trials that were rewarded, without altering the number of trials that animals failed. This resulted in an overall shift in the proportion failed trials that was specific to which reward was being pursued. Although the number of trials that animals failed was unchanged after hormone treatment, the animal’s behavior during failed vs. completed trials did differ based on reproductive status.

Increased motivation for each reward was driven by an increase in responding during completed trials. During failed trials, animals show no changes in the number of responses for the active reward as a consequence of hormone treatment. However, during both pellet and mate trials, hormone primed, but not unprimed, failed trials were characterized by an increase in the number of responses that animals made for the alternate reward.

### Hormone Priming Biases Attention for Reward Paired Cues

In addition to changes in instrumental responding for each reward, the shift in preference for food vs. sex was apparent in what elements of the apparatus animals attended to during the task. There was no effect of hormone priming on the amount of time animals spent interacting with elements of the apparatus associated with access to the mate, including the mate paired light cue and door. However, females spent significantly less time interacting with the food tray and the food paired cue after hormone treatment. Within the female side of the chamber, where all of the response elements are located, animals have limited options for what to direct their attention toward. The decrease in time spent focused on the food associated elements, without a concurrent increase in time spent focused on the mate paired elements, indicates that hormone primed females are instead increasing the amount of time they spend engaging in some third behavior. One possibility is that the animals are waiting for the desired time period between intromissions to elapse before returning the male side. As mentioned previously, females will actively pace the rate of copulation when given the opportunity. The length of the interval between intromissions is dependent on the intensity of stimulation and is necessary for the induction of the progestational reflex required for successful implantation of a fertilized embryo as well as sexual reward (Erskine et al., 1989, 2004; Jenkins and Becker, 2003b). DA release during female sexual behavior rises during the time leading up to, but not during intromissions (Jenkins and Becker, 2003a). This may indicate that this waiting period, rather than being a passive phase in between bouts of sexual behavior, is instead an active behavior that is important for the rewarding aspects of the female sexual experience.

Operant responding on the fixed interval was maintained by the contingent presentation of a reward paired light cue. During reward learning, increases in DA release shifts from the presentation of the primary reinforcer to the predictive cue (Day et al., 2007). In addition to facilitating learning, this shift in DA release has been proposed to mediate the incentive salience of reward paired cues (Berridge, 2007). In the current paradigm, which allows us to distinguish between changes in consummatory behaviors and changes in appetitive reward-seeking, changes in operant responding during each trial can be interpreted as changes in the incentive value of the reward-paired cue. This is supported by findings that response rates are altered during trial by trial changes in motivational state (e.g., following ejaculation). Within this model, the ability of ovarian hormones to increase responding for one reward, while simultaneously reducing responding for another, suggests that induction of sexual receptivity can selectively alter the incentive salience of reward paired cues, thereby directing motivated behaviors in pursuit of specific rewards.

## Conclusion

Understanding how organisms balance motivations for competing rewards is key to understanding how motivation influences decision making. The majority of research evaluates motivation for a reward when only one reward is available, and while this approach can be helpful in understanding the neural circuitry underlying motivation, it does not help us to understand how these processes are integrated during adaptive decision making. In our paradigm, female rats first choose between two available rewards, then make a variable number of responses to indicate how motivated they are for each reward. This allows us, for the first time, to measure how concurrent changes in feeding and sexual behavior, during periods of sexual receptivity are reflected by changes in their motivation for both rewards simultaneously. These findings demonstrate experimentally that changes in motivation for food after hormone treatment act to enhance motivation for sex. When given the opportunity to choose between sex and food, OVX female rats show a preference for food reward that is reversed following administration of EB and P. This shift in the choice between food and sex is reflected by concurrent changes in their motivation for each reward, as measured by operant responding on a FI schedule. We propose that these findings provide experimental evidence for the ultimate or adaptive purpose of periovulatory changes in feeding behavior. While scholars have speculated that seemingly paradoxical reductions in food intake and body weight during ovulation reflect a shift in the female’s behavioral priorities, this hypothesis remained untested. The paradigm described herein allows us to disentangle the effects of ovarian hormones on motivation from their effects on consummatory aspects of feeding and reproductive behavior. The results presented here demonstrate the adaptive value of periovulatory changes in feeding behavior.

## Data Availability Statement

The datasets generated for this study are available on request to the corresponding author.

## Ethics Statement

The animal study was reviewed and approved by University of Michigan Institutional Animal Care and Use Committee.

## Author Contributions

KY collected and analyzed the data. KY, JC, and JB designed the experiments and wrote the manuscript.

## Conflict of Interest

The authors declare that the research was conducted in the absence of any commercial or financial relationships that could be construed as a potential conflict of interest.
