# Supplementary material for: Ovarian Hormones Mediate Changes in Adaptive Choice and Motivation in Female Rats
Source: Front Behav Neurosci. 2019 Nov 12;13:250. doi: 10.3389/fnbeh.2019.00250 (PMC6861187; doi:10.3389/fnbeh.2019.00250)
Supplement: Supplementary file 1 [file Data_Sheet_1.PDF]

Table 1. Two-way ANOVA statistics

Measure	Trial Type	Treatment	Treatment x Trial Type
**# of Trials Initiated**<br>Figure 2B	*F*(1,7)=1.34, *p*=0.28	***F*(1,7)=19.28, *p*<0.01**	***F*(1,7)=7.30, *p*=0.03**
**% of Total Trials**<br>Figure 2C	*F*(1,7)=0.18, *p*=0.68	*F*(1,7)=0.00, *p*>0.99	***F*(1,7)=22.20, *p*<0.001**
**% of Total Trials Compared by Trial Outcome**<br>Figure 2D	***F*(3,28)=3.75, *p*=0.02**	*F*(1,28)=0.00, *p*>0.99	***F*(3,28)=13.79, *p*<0.0001**
**% Failed**<br>Figure 2E	*F*(1,27)=0.86, *p*=0.36	*F*(1,27)=0.3853, *p*=0.54	***F*(1,27)=4.88, *p*=0.04**
**Average Time (s) per Trial**<br>Figure 3A	***F*(1,7)=11.43, *p*=0.01**	***F*(1,7)=7.11, *p*=0.32**	*F*(1,7)=0.76, *p*=0.41
**Total Time (s)**<br>Figure 3B	*F*(1,7)=2.20, *p*=0.18	*F*(1,7)=0.64, *p*=0.45	***F*(1,7)=25.16, *p*<0.01**
**Active Responses/Fixed Interval**<br>Figure 4A	*F*(1,7)=2.37 *p*=0.17	*F*(1,7)=0.10, *p*=0.77	***F*(1,7)=11.44, *p*=0.01**
**Inactive Responses/Fixed Interval**<br>Figure 4B	*F*(1,7)=0.96, *p*=0.36	*F*(1,7)=1.06, *p*=0.33	*F*(1,7)=1.80, *p*=0.22
**Active Mate Responses/Fixed Interval Compared By Trial Outcome**<br>Figure 4C	***F*(2,14)=19.18, *p*<0.0001**	*F*(1,7)=4.20, *p*=0.08	***F*(2,14)=6.68, *p*<0.01**
**Active Pellet Responses/Fixed Interval**<br>Figure 4D	***F*(2,14)=8.10, *p*<0.01**	*F*(1,7)=4.34, *p*=0.08	*F*(2,14)=1.23, *p*=0.32
**Inactive Mate Responses/Fixed Interval Compared by Trial Outcome**<br>Figure 4E	***F*(2,14)=4.40, *p*=0.03**	*F*(1,7)=0.24, *p*=0.64	*F*(2,14)=0.35, *p*=0.71
**Inactive Pellet Responses/Fixed Interval Compared by Trial Outcome**<br>Figure 4F	***F*(2,14)=4.67, *p*=0.03**	*F*(1,7)=1.38, *p*=0.28	*F*(2,14)=0.18, *p*=0.84

Significant effects shown in bold.
